# Supplementary material for: Development and evaluation of multiepitope fusion proteins for serological diagnosis of animal brucellosis
Source: Microbiol Spectr. 2025 Sep 30;13(11):e00516-25. doi: 10.1128/spectrum.00516-25 (PMC12584648; doi:10.1128/spectrum.00516-25)
Supplement: Supplemental figures — Fig. S1 and S2. [file spectrum.00516-25-s0003.docx]

**Supporting information 3:** Original images for SDS-PAGE of fusion protein 1 and fusion protein 2. Amino Acid Sequence of fusion protein 1 and fusion protein 2.

Amino Acid Sequence of Fusion Protein 1:

NQMTTQPARIAVGGGSQPIYVYPDDKNNLKEPTITGGGSRPPMPMPIARGGGGSAPDNSVPIAAGENSYNVSVVNVVFEGGGSNLDNVSPPPPPAPVNAVPASTVGGGSKGNLDSPTQFPNAPSTDMSAQSGTQVGGGSAFAPDLTPGGGGSQTKYGQGYGGGSQGRFDGQTTGGGGGSNLPNNAGDLGLGGGSQPPVPAPVEVGGGSTSTVGSIKPGGGSNGLDDESGGGSNNSRHDGQYGDFSDDRDVADGGVSGGGSGGEDVDNDGGGSSSAATPNQNYGQWGGGGSTKFGGEWKDTVGGGSKGGDDVYSGTDRNGWDGGGSNNSGVDGKYGNETSSGGGGSNDGGYTGTTNYHIGGGSPDQNYGQWGGGGGSVSYIKFGGEWKNTVAEDNGGGSVSEPSAPTAAPGGGSFDKEDNEQVSGGGSQAGYNWQLDNGVVLGAGGGSGDDASALHMWGGGSEKLTPGYGGGSSCAPGEKDGKIVPGGGSNTHHHLGPEGDGHMGGGGSPHLKKLGGGSMVHVGGDNYSDKPEPLGGGSRRYDDPMVTKDKDLVPGGGSGKEPHKGVNGGGSTRLIERNTTIPTKKSGGGSIPPAPRGVGGGSDKGTGKEHQGGGSDAEANAEADKKRRESVEAKNGGGSEGAGAEGGEQASSSKDDVVDGGGSSKYEFPGDGGGSIPTPERPIGGGSDEGGRHTPFFTGGGSGTEMVMP


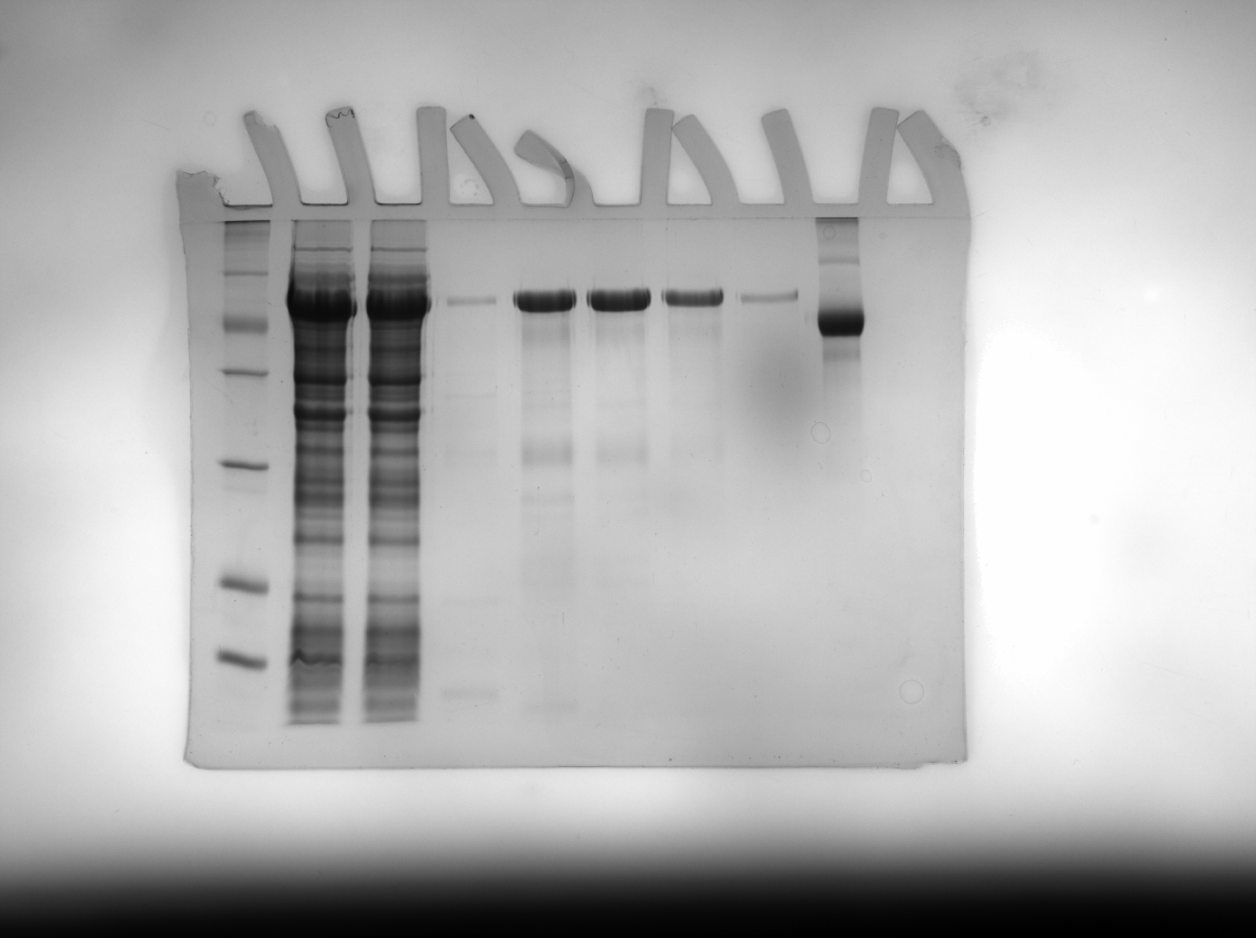


**Figure S1. Original image for fusion protein 1.**

Amino Acid Sequence of Fusion Protein 2:

TMLAAAPDNSVPIAAGENSYNVSVNVVFEIKGGGSKKAGIEDRDLQTGGINIQPIYVYPDDKNNLKEPTITGYGGGSSSKDDVVDADYEEIDDNKKSSGGGSQTREHILGGGSVIEEWAAKVRGDVNITDQFSVWLQGAYSSAATPDQNYGQWGGGGSSWTGGYIGINAGYAGGKFKHPFSSFDKEDNEQVSGSLDVTAGGFVGGGSQAGYNWQLDNGVVLGAGGGSMVYGTGGLAYGKVKSAFNLGDDAPALHTWSDKTKAGWTLGAGAEGGGSEYLYTDLGKRNLVDVDNSFLGGGSAPGEKDGKIVPAGGGS LAEIKQRSLMVHVGGDNYSDKPEPLGG

**
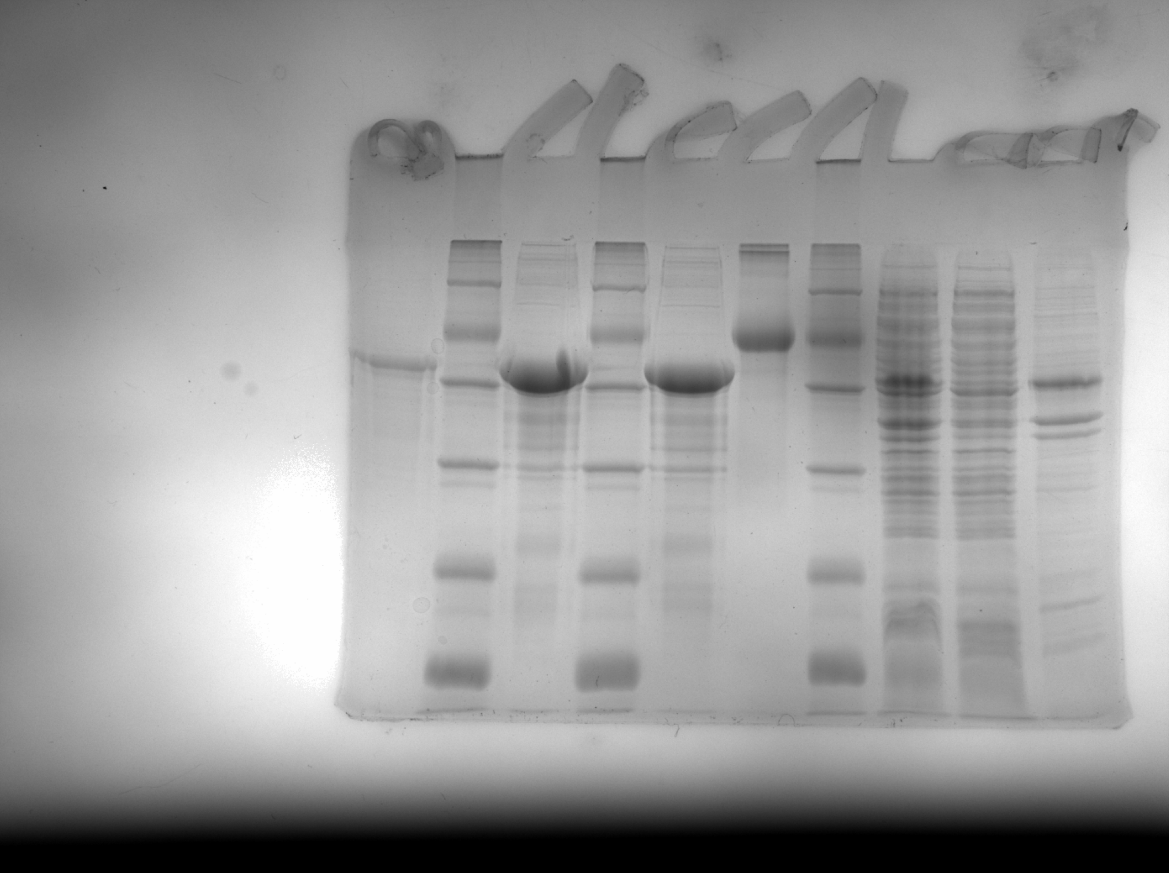
**

**Figure S2. Original image for fusion protein 2.**
