# Supplementary material for: Development and evaluation of multiepitope fusion proteins for serological diagnosis of animal brucellosis
Source: Microbiol Spectr. 2025 Sep 30;13(11):e00516-25. doi: 10.1128/spectrum.00516-25 (PMC12584648; doi:10.1128/spectrum.00516-25)
Supplement: Supplemental material — Protocols for endotoxin removal and endotoxin detection; Table S1. [file spectrum.00516-25-s0004.docx]

**Supporting information 1:** Protocols for endotoxin removal and endotoxin detection.

**1. Protein Sample Endotoxin Removal Procedure, Protein Endotoxin Removal Kit (Beyotime, C0268S).**

a. Transfer 500μL of the protein sample to be processed into a 1.5mL endotoxin-free centrifuge tube.

b. Add 100μL of Endotoxin Removal Solution A, vortex to mix thoroughly. The solution will become turbid.

c. Add 120μL Endotoxin Removal Solution B and mix. The solution remains turbid.

d. Add 1μL Separation Indicator and mix.

e. Incubate at 4°C or on ice for 5 minutes. The solution becomes clear.

f. Incubate at 37°C for 1 minute. The solution exhibits a distinct milky white turbidity.

g. Centrifuge at 25°C or room temperature at 12,000–14,000g for 5 minutes to achieve biphasic separation. The endotoxin-removed protein will reside in the upper aqueous phase, while endotoxins will form an organic phase at the tube bottom.

h. Promptly and carefully aspirate the upper aqueous phase into a new endotoxin-free 1.5mL centrifuge tube. Discard the organic phase.

**2. Endotoxin Detection Kit (Limulus Reagent Dynamic Turbidimetric Method) (Beyotime, C0271S)**

a. Standard Preparation: Take 100 EU of endotoxin standard, add 1 mL of deionised water to prepare a final concentration of 100 EU/mL. Perform a 10-fold serial dilution to prepare four concentrations: 10 EU/mL, 1 EU/mL, 0.1 EU/mL, and 0.01 EU/mL. Dispense 50 µL per well into a 96-well microplate (Beyotime, FULA961).

b. Sample Preparation: Transfer the test sample into a 96-well microplate (Beyotime, FULA961), dispensing 50 µL per well.

c. Sample assay: Add 50 µL of endotoxin assay reagent solution to each well containing standards and test samples. After gentle shaking to ensure thorough mixing, measure the absorbance at 340 nm. Continuously monitor absorbance values dynamically (every 60 seconds). Set the initial OD value to 0.02. Plot a standard curve of reaction initiation time T versus endotoxin concentration C (fit the log_10_T versus log_10_C curve).

Calculations indicated that the standard curve is given by log_10_T = -0.430 log_10_C + 3.154, R^2^=0.971.

Table S1 Endotoxin test results for fusion protein 1 and fusion protein 2.

| Proteins | T(s) | |
| --- | --- | --- |
|  | Before endotoxin removal | After endotoxin removal |
| Fusion protein 1 | 360 | 3420 |
| Fusion protein 2 | 420 | 4140 |
